# The protocol for a randomised controlled trial comparing intermittent and graded exercise to usual care for chronic fatigue syndrome patients

**DOI:** 10.1186/2052-1847-5-16

**Published:** 2013-08-30

**Authors:** Suzanne Broadbent, Rosanne Coutts

**Affiliations:** 1 School of Health and Human Sciences, Southern Cross University, PO Box 157, Lismore, NSW 2480, Australia

**Keywords:** Chronic fatigue syndrome, Interval training, Graded exercise, Immune function, Lymphocyte function

## Abstract

**Background:**

Chronic Fatigue Syndrome is a debilitating disorder with an unknown aetiology but suspected multifactorial origins. Common “triggers” include severe viral infections and emotional stress. Recent studies have also found evidence of immune dysfunction and elevated inflammatory cytokines in CFS patients, but there has been considerable variation in the outcome measures and magnitude of these studies. Currently, there is no cure for CFS but treatments include rest, specialist medical care, cognitive behavioural therapy, and graded (self-paced) exercise. To date, several studies have examined the efficacy of graded exercise with or without Cognitive Behavioural Therapy, with some success for patients. However, improvements in functional capacity have not necessarily correlated with improvements in immune function, fatigue or other symptoms. This 12-week pilot trial compares graded and intermittent exercise to normal care, measuring physiological outcomes, fatigue levels, immune function and wellness.

**Methods/design:**

90 patients aged between 16 to 60 years, who meet the diagnostic criteria for CFS and have been diagnosed by their medical practitioner, will be randomly recruited into groups consisting of Intermittent exercise, Graded exercise and usual care (Control). The outcomes will be measured pre-study (Week 0) and post-study (Week 13). Primary outcomes are VO_2peak_, anaerobic threshold, peak power, levels of fatigue, immune cell (CD3^+^CD4^+^, CD3^+^CD8^+^, CD19^+^, CD 16^+^CD56^+^) concentrations and activation. Secondary outcomes include onset of secondary CFS symptoms (e.g. fever, swollen lymph nodes), wellness, mood and sleep patterns. Primary analysis will be based on intention to treat using logistic regression models to compare treatments. Quantitative data will be analysed using repeated measures ANOVA with a linear model, and Cohen’s effect size. Qualitative data such as participants’ responses (e.g. changes in mood and other reactions) following the exercise modalities will be read and sections demarcated. A code will be applied to each segment. A prevalence of codes will be considered thematically.

**Discussion:**

The results of the trial will provide information about the efficacy of intermittent and graded exercise compared to usual care (rest and lifestyle recommendations), contributing to the evidence for best-practice CFS management.

**Trial registration:**

Australia and New Zealand Clinical Trials Registry ACTRN12612001241820.

## Background

Currently there is no clear aetiology associated with Chronic Fatigue Syndrome (also known as Myalgic Encephalitis). The syndrome is characterised by persistent, disabling and/or recurring fatigue, not alleviated by rest, and with other symptoms such as muscle weakness and pain, swollen lymph nodes and fever, poor concentration and reduced quality of life [1,2]. A medical diagnosis is made when three of the following criteria from the Centre for Disease Control and Prevention’s modified case definition of CFS, have been met [3]:

(1) The individual has had severe chronic fatigue for 6 or more consecutive months and the fatigue is not due to ongoing exertion or other medical conditions associated with fatigue (these other conditions need to be ruled out by a doctor after diagnostic tests have been conducted).

(2) The fatigue significantly interferes with daily activities and work.

(3) The individual concurrently has 4 or more of the following 8 symptoms: post-exertion malaise lasting more than 24 hours; unrefreshing sleep; significant impairment of short-term memory or concentration; muscle pain; pain in the joints without swelling or redness; headaches of a new type, pattern, or severity; tender lymph nodes in the neck or armpit; a sore throat that is frequent or recurring.

These symptoms should have persisted or recurred during six or more consecutive months of illness and they cannot have pre-dated the fatigue.

CFS is often triggered by a severe viral infection, such as Epstein Barr virus [4]. There is also increasing evidence to suggest that CFS is also associated with immune system dysfunction, such as increased inflammatory cytokines and reduced lymphocyte activation [4-7]. CFS can cause severe interruptions to education or employment for patients, and the physical inactivity associated with the syndrome may increase the risk of other chronic conditions such as cardiac disease, metabolic conditions and cancer. Earlier studies found that the majority of individuals with CFS have slight to substantial improvement within 1.5 - 4 years with less than 10% reporting complete recovery [8,9]. Approximately 20-50% of adults with CFS demonstrate some improvement in the condition, although only 6% returned to levels of normal function [10,11]. Rehabilitation is usually slow with symptoms described as fluctuating. Approximately 10% of individuals with CFS may improve for periods of at least a year, with a proportion relapsing intermittently [10,11]. Most patients relate that they never return to their pre-morbid level of health [11,12]. After a period of rest, some individuals may return to work but usually with limited working hours. It is apparent that the combination of tiredness, poor memory and difficulties with concentration are reflected in the changes to their productivity.

CFS patients are often advised to remain as active as possible but patients usually show a range of physiological responses to exercise indicative of fatigue and deconditioning. Such responses include reduced exercise capacity, early onset of anaerobic threshold, increased lactate responses during incremental exercise, increased heart rate responses, and also anomalies within perception of their physical effort such as higher rates of perceived exertion compared to controls [2,13,14]. Hypothalamic, mitochondrial and sympathetic dysfunction may also be implicated in exercise and fatigue responses in CFS patients [3,15,16].

Strategies to manage CFS include pharmacology, physical activity where appropriate (for example graded exercise (GE) and pacing), and psychological approaches such as cognitive behavioural therapy (CBT) which seek to modify behaviour, beliefs and coping strategies. [17-20]. Current best practice for the physical training of CFS patients involves GE sessions where the patient exercises at a well-tolerated constant rate or load (steady state), and the duration of the exercise session is gradually increased over time [2,18-20]. CBT is usually combined with exercise management [18,20].

Evidence of the efficacy of GE, CBT, pacing and other prescriptions of physical activity has been mixed. Some CFS patients still report symptom exacerbation and muscle pain with GE, and consequently are less likely to be physically active [21,22]. Yet other studies have reported that aerobic and resistance exercise improves aerobic capacity and strength [13], fatigue [17], profile of mood states and inflammatory cytokines [14,17,21,23-26].

Intermittent training, where intervals of exercise are alternated with intervals of rest or very low intensity exercise, has been used with cardiac [27], pulmonary [28,29], cancer [30] and older clients who are very deconditioned [31]. Clapp et al. (1999) reported that an acute session of intermittent exercise for 30 minutes did not exacerbate CFS symptoms immediately post-exercise, or for up to 7 days post exercise [32]. In CFS patients, intermittent training may increase exercise tolerance by decreasing perceived exertion, and delaying the onset of fatigue and muscle pain, whilst still providing the same total amount of physical activity as GE. Patients may find it easier to adhere to an intermittent exercise programme if their symptoms are not exacerbated, and therefore may improve their health.

Study hypotheses and aims: the primary aim of the trial is to investigate the efficacy of 12 weeks of intermittent or graded exercise on aerobic capacity, fatigue, immune function and wellness in CFS patients compared to standard care (typically rest, pharmacology and advice about physical activity). We hypothesise that:

1) Both exercise interventions will improve exercise tolerance, aerobic capacity and wellness compared to the control condition, and

2) IE will result in greater exercise tolerance, less fatigue and other symptoms, improved immune function and better exercise adherence compared to GE.

## Methods

### Design

This study is a randomised controlled trial that will compare the outcomes of a 12-week intermittent cycling exercise program to a graded exercise cycling program and standard care (rest and lifestyle advice). The primary outcomes of the study are aerobic capacity (VO_2peak_), perceived exertion, fatigue, muscle pain, immune cell counts, lymphocyte (CD4^+^, CD8^+^, CD19^+^, CD16^+^56^+^) concentrations and function. Secondary outcomes are wellness indices and physiological adaptations to exercise (changes in heart rate, heart rate variability, blood pressure, body composition, anaerobic threshold) and measures of presence, severity and type of fatigue (mental and physical). Southern Cross University Research Ethics Committee has approved all study procedures (HREC ECN-13-066).

### Sample size and power calculation

A previous exercise intervention study provided VO_2peak_ data from a CFS sedentary, age-matched cohort (16.2±1.3 yr) [18]. This data has been used to compute statistical power *a priori* using the non-commercial statistical power analysis program G*Power. The control group is expected to experience no change in the aerobic capacity after 12-weeks while the intervention groups are expected to increase this measure following the exercise interventions. Setting an alpha level of 0.05, approximately 75 participants (25 per group) will provide 82% power to detect a statistically significant difference between groups. Recruitment will be inflated to 90 participants to enable a 20% participant attrition rate.

### Participants

The inclusion criteria for the study are a medical diagnosis of CFS from the participant’s medical practitioner, according to CDC criteria; an age range of 16 to 60 yr; no diagnosed cardiorespiratory, endocrine, metabolic condition or current musculoskeletal injury that would make exercise participation hazardous; the ability to communicate in English; provision of informed consent; willingness to participate in three exercise sessions per week. Participants will be recruited from the local community through advertisements at the Southern Cross University campus and Health Clinic, local medical clinics and hospitals, local newspapers, television and radio media. Participants will be randomized *via* computer-generated randomly permuted blocks stratified by gender and age (< 40 years; > 40 years) into the intervention groups and control group. A university employee not involved in exercise testing or delivery of the intervention will prepare the randomization assignments. Group assignment will be delivered to participants in sealed envelopes upon the completion of baseline testing. Participants who have been randomized into the control group will be offered a supervised 12-week exercise program upon completion of the study.

### Interventions

#### Exercise groups

Participants randomized to the exercise intervention groups will engage in a 12-week exercise program consisting of either intermittent exercise (IE) or graded exercise (GE). The exercise sessions will be group-based and conducted at the Southern Cross University Health Clinic, three times per week. All sessions will be supervised by an accredited Exercise Physiologist (AEP) and post-graduate clinical exercise physiology students. Each exercise session will consist of a 5 minute gentle warm up of unloaded cycling, followed by initially a 10 to 15 minute block of either steady state exercise (GE) or intermittent exercise (IE) at an intensity pre-determined from the baseline VO_2peak_ cycle test for each participant. Depending upon the symptom limits of the exercise, the duration of the exercise block can be gradually increased for each participant as tolerated to either a steady state (constant effort) low-moderate intensity cycling period, (50% VO_2peak_, RPE 3 [0–10 Borg Scale]) initially for 10 minutes (GE group) OR an intermittent exercise block of 1 minute of moderate intensity cycling (60% VO_2peak_, RPE 4–5) alternated with 1 minute of unloaded or very low intensity/unloaded cycling (20-30% VO_2peak_, RPE 1–2), totalling 20 minutes. A cool down of 5 minutes unloaded cycling plus stretching of main muscle groups for both groups is also included in the exercise session.

During the 12 weeks of training, we aim to progress the duration of GE towards 20 min, as tolerated by the participant, and to progress the IE participants towards intervals of 2–3 min of moderate intensity cycling, alternated with 1 minute intervals of low intensity cycling, totalling 25–30 min duration. Resting heart rate, heart rate variability (standard deviation of the normal to normal R-R intervals and the root mean square of differences of successive R-R intervals), resting blood pressure, RPE, (0–10 Borg Scale) and fatigue levels will be recorded prior to, and after, each exercise session. Participants will be asked to record any changes, such as elevated or depressed mood, changes to sleep patterns or general well-being, that they perceive to have occurred during their participation in the exercise training.

#### Control group

Participants randomized to the control group will be asked to follow the advice of their medical practitioner (rest, maintaining activity for daily activities, [ADL]). Control group participants will be advised not to engage in any other physical activity, and will be offered a similar 12-week exercise program after completion of the current study. All study participants will be asked to complete an ADL diary to control for normal daily movements.

### Outcome measures

All outcome measures will be collected at baseline (Week 0) and after the exercise interventions (Week 13). Trained research personnel blinded to the randomization procedures will conduct the initial participant screenings and baseline assessments, which include a full medical history, resting heart rate, resting blood pressure and anthropometry (height; weight; BMI; waist, hip and limb girths), fatigue severity and wellness questionnaires. The project outcome measures consist of a full cell count; lymphocyte receptor flow cytometric assays; incremental cycling test (VO_2peak,_ anaerobic threshold); spirometry; Personal Well Being Index [33] and Fatigue Severity Scale [34].

#### Measures of immune function

The baseline and post-study full blood count with leukocyte differential will be performed by Northern Pathology at Lismore Base Hospital. Blood samples will be de-identified and numerically coded. Ten mL of blood will also be drawn to provide for flow cytometric assays of CD3^+^CD4^+^CD^+^135 (T helper), CD3^+^CD8^+^ (cytotoxic effector) lymphocytes, CD45^+^ (HLA marker), CD3^+^CD19^+^ (B cells) and CD56^+^CD16^+^ Natural Killer (NK) cells. The absolute concentration of these lymphocyte subsets will be measured by flow cytometer (FACSCanto ll, BD Biosciences, Australia) using the BD Multitest 6-colour direct immunofluorescence TBNK reagent and BD Trucount tubes (BD Biosciences, Australia) and FACSDiva v4.1 software (BD Biosciences, Australia).

The method for stimulating and measuring CD4^+^CD25^+^CD134^+^ expression in whole blood samples is described by Zaunders et al., (2009) [35]. Briefly, sodium heparin anti-coagulated whole blood (0.5 mL) will be mixed with 0.5 mL RPMI-1640 (Sigma-Aldrich, Australia), and will be stimulated by 5 μg/mL phytohemagglutinin (PHA) (Sigma-Aldrich, Australia) for 48 hr at 37°C in a 5% CO_2_ incubator. After culture, 100 μL of sample will be stained with the following conjugated fluorochromes for 15 min at room temperature (CD3-FITC; CD4-Per-CP; CD25-APC; CD134-PE, BD Biosciences, Australia), followed by washing in PBS (1 mL), resuspension in 0.5 mL of 0.5% paraformaldehyde (Sigma-Aldrich, Australia) and then multi-parameter flow cytometric analysis.

In a separate assay, both CD3^+^CD8^+^ and NK lymphocytes can be stimulated in an anti-coagulated whole blood sample (0.5 mL blood and 0.5 mL RPMI-1640) by using phorbol-12-myristate-13-acetate (PMA) (2.5 μg/mL) and ionomycin (0.5 μg/mL). The activation of CD3^+^CD8^+^ will be assessed through the co-expression of CD38 in response PMA and ionomycin following a 6 hr incubation at 37°C in a 5% CO_2_ incubator. The cytotoxic response of CD3^+^CD8^+^ will be measured with the expression of LAMP-1 (CD107a) and LAMP-2 (107b) as positive markers of degranulation (perforin-granzyme mediated killing) plus the concurrent intracellular production of IFN-γ [36]. NK lymphocytes will be also be stimulated by PMA and ionomycin for 6 hr to measure the expression of CD107a as an indicator of cytotoxic activity and degranulation [37]. After stimulation of CD3^+^CD8^+^ and NK cells, washing of the cells in PBS and resuspension in paraformaldehyde, receptors can be measured as follows: CD3-Per-CP; CD8-APC-H7; CD38-PE-Cy7; CD107a-APC; CD107b-FITC; CD16-PE; CD56-PE (BD Biosciences, Australia). In each experiment, a negative control (no PHA or PMA/ionomycin) will be included to control for spontaneous receptor expression or cytokine production.

#### Measures of aerobic capacity

Each participant will undergo an incremental test to volitional exhaustion on a cycle ergometer (Lode Excalibur, Holland). Peak *V*O_2_ (mL.kg^-1^.min^-1^), *V*CO_2_, ventilation (*V*_E_ BTPS L min^-1^), ventilatory equivalents (*V*E/*V*O_2_, *V*E/*V*CO_2_) and respiratory exchange ratio (RER) will be measured using open circuit spirometry (AEI, Australia), and recorded every 15 s, with peak values determined from the average of the two highest values attained over two collection periods during the exercise test. The criteria for attaining *V*O_2peak_ are when (1) *V*O_2_ increases by < 0.15 L. min^-1^ despite an increase in power, (2) heart rate is within ten beats of age-predicted maximum, (3) when RER ≥ 1.15 and (4) when RPE ≥ 8 on the 0–10 Borg Scale. However we also accept that symptom limits (fatigue) may stop the exercise test before these criteria are reached. Throughout each incremental exercise test, heart rate and rhythm will be monitored from bipolar leads in the CM5 position; blood pressure will be monitored every 2 min with standard auscultation; power (W) will be recorded every 60 s (Lode Excalibur, Groningen, Holland). Anaerobic threshold will be determined from incremental test results using the V slope method [38], a computerized regression analysis of the slopes of the CO_2_ uptake (VCO_2_) vs. O_2_ uptake (VO_2_) plot, which detects the beginning of the excess CO_2_ output generated from the buffering of [H^+^], relative to oxygen uptake.

#### Fatigue and muscle pain

RPE and fatigue will be recorded at rest and during the incremental exercise test. Participants will be asked to complete a linear pain scale (0 – 10) to indicate if they have any muscle or joint stiffness or soreness prior to the exercise test, immediately post-exercise and also 24 hours post-exercise test. RPE and pain scales will also be completed at every training session to carefully monitor symptoms. The Fatigue Severity Scale [34] and the Chalder Fatigue Scale [39], both CFS-specific, will be administered pre- and post-study to assess the severity and nature of the fatigue, mental and physical.

#### Wellness indices

Quality of life and wellness will be assessed pre- and post-study using the Personal Well Being Index, [33] and the Revised Clinical Interview Schedule medical history [40].

### Statistical analyses

Primary analysis will be via intention-to-treat with all patients included regardless of dropout or level of adherence. Missing data will be imputed according to the maximum likelihood expectation algorithm via the Statistical Package for the Social Sciences (SPSS© Version 19.0). Physiological and exercise test data will be presented as the mean ± standard deviation, with confidence intervals used to express group differences. Changes between groups and the time effect for each group will be determined by repeated measures analysis of variance with a Bonferroni adjustment for significant differences between groups. Cohen’s Effect sizes and 90% confidence intervals will be calculated, to utilise a magnitude-based approach to inferences [41], with the following descriptors applied: (1) Effect size thresholds: <0.2 trivial, <0.6 small, <1.2 moderate, <2.0 large, <4.0 very large, >4.0 extremely large, and (2) Thresholds for assigning qualitative terms to chances of substantial effects: <0.5%, almost certainly not; <5.0%, very unlikely; <25%, unlikely; <75%, possible; >75%, likely; >95%, very likely; >99.5%, almost certain. An effect is unclear if its confidence interval includes both substantial increases and decreases [41]. A p value of < 0.05 will be considered indicative of statistical significance; clinical significance will be interpreted in light of the meaningfulness and magnitude of the adaptations observed. Data from linear pain scales will be recorded pre- and post-study, and reported as frequencies of scores between 0 and 10. Data will then be analysed using ANOVA (group and time effects). Data from wellness indices will be recorded pre and post, combined into domains and a representative total score from the time points analysed using an ANOVA. Data (non parametric) from the Fatigue scales, also measured at time points, will be collated and analysed using a Kruskall-Wallis test.

## Discussion

The results of this study will contribute evidence to best-practice for management of CFS. Findings from this research could improve health outcomes by reducing fatigue, increasing aerobic capacity, wellness and immune responses. Desired research outcomes could also be applied to the management of other chronic health conditions where fatigue is a contributing factor.

## Competing interests

The authors declare that they have no competing interests.

## Authors’ contributions

SB designed the training study, physiological and immunological testing procedures and drafted the manuscript. RC provided the wellness questionnaire and fatigue scales. SB and RC provided consultation regarding statistical analyses. Both authors have read and approved the manuscript.

## Pre-publication history

The pre-publication history for this paper can be accessed here:

http://www.biomedcentral.com/2052-1847/5/16/prepub
